# A Simple and Effective Method for Solid Medium Cultivation of Strictly Hydrogen- and Sulfur-oxidizing Chemolithoautotrophs Predominant in Deep-‍sea Hydrothermal Fields

**DOI:** 10.1264/jsme2.ME23072

**Published:** 2023-12-16

**Authors:** Hisashi Muto, Junichi Miyazaki, Shigeki Sawayama, Ken Takai, Satoshi Nakagawa

**Affiliations:** 1 Laboratory of Marine Environmental Microbiology, Division of Applied Biosciences, Graduate School of Agriculture, Kyoto University, Oiwake-cho, Kitashirakawa, Sakyo-ku, Kyoto 606–8502, Japan; 2 Institute for Extra-cutting-edge Science and Technology Avant-garde Research (X-star), Japan Agency for Marine-Earth Science & Technology (JAMSTEC), 2–15 Natsushima-cho, Yokosuka 237–0061, Japan; 3 Deep-Sea and Deep Subsurface Life Research Group, Exploratory Research Center on Life and Living Systems (ExCELLS), National Institutes of Natural Sciences (NINS), 5–1 Higashiyama Myodaiji, Okazaki 444–8787, Japan

**Keywords:** deep-sea hydrothermal field, chemolithoautotrophy, cultivation, solid media, colony formation

## Abstract

Strictly hydrogen- and sulfur-oxidizing chemolithoautotrophic bacteria, particularly members of the phyla *Campylobacterota* and *Aquificota*, have a cosmopolitan distribution in deep-sea hydrothermal fields. The successful cultivation of these microorganisms in liquid media has provided insights into their physiological, evolutionary, and ecological characteristics. Notably, recent population genetic studies on *Sulfurimonas* (*Campylobacterota*) and *Persephonella* (*Aquificota*) revealed geographic separation in their populations. Advances in this field of research are largely dependent on the availability of pure cultures, which demand labor-intensive liquid cultivation procedures, such as dilution-to-extinction, given the longstanding assumption that many strictly or facultatively anaerobic chemolithoautotrophs cannot easily form colonies on solid media. We herein describe a simple and cost-effective approach for cultivating these chemolithoautotrophs on solid media. The results obtained suggest that not only the choice of gelling agent, but also the gas phase composition significantly affect the colony-forming ratio of diverse laboratory strains. The use of gellan gum as a gelling agent combined with high concentrations of H_2_ and CO_2_ in a pouch bag promoted the formation of colonies. This contrasted with the absence of colony formation on an agar-solidified medium, in which thiosulfate served as an electron donor, nitrate as an electron acceptor, and bicarbonate as a carbon source, placed in anaerobic jars under an N_2_ atmosphere. Our method efficiently isolated chemolithoautotrophs from a deep-sea vent sample, underscoring its potential value in research requiring pure cultures of hydrogen- and sulfur-oxidizing chemolithoautotrophs.

Deep-sea hydrothermal vents are host to remarkably productive ecosystems fueled primarily by chemolithoautotrophic microorganisms. These microorganisms utilize chemical energy from inorganic compounds in vent fluids to convert CO_2_ into organic compounds (Nakagawa and Takai, 2008). Sulfur- and/or hydrogen-oxidizing chemolithoautotrophs, particularly members of the phyla *Campylobacterota* and *Aquificota*, are widespread in these ecosystems, playing essential roles in carbon, sulfur, and nitrogen cycles (Reysenbach *et al.*, 2000; Nakagawa *et al.*, 2005a, 2005b; Takai *et al.*, 2005; Ferrera *et al.*, 2007; Vetriani *et al.*, 2014). To obtain a more detailed understanding of their physiological and ecological characteristics, pure cultures of these microbes have been obtained from deep-sea vents worldwide, generally employing liquid media and dilution-to-extinction methods. Although a limited number of strains have been derived using agar- or gellan gum-solidified media (L’Haridon *et al.*, 1998; Gotz *et al.*, 2002; Miroshnichenko *et al.*, 2002; Inagaki *et al.*, 2003; Voordeckers *et al.*, 2005; Perez-Rodriguez *et al.*, 2010; Francois *et al.*, 2020), the effects of various factors, such as gelling agents, medium preparation procedures, and incubation conditions, on these microorganisms remain unclear. This suggests the potential to further enhance the efficacy of solid medium cultivation strategies for deep-sea vent chemolithoautotrophs.

Solid medium cultivation offers several advantages over liquid medium cultivation. It simplifies the isolation and maintenance of environmental microorganisms via colony formation, facilitating the simultaneous isolation of multiple strains from a single sample. The choice of gelling agent significantly affects microbial growth on solid media. Although commonly used in standard microbiology experiments, agar is known to inhibit the growth of a wide range of microorganisms (Tuovinen and Kelly, 1974; Tanaka *et al.*, 2014; Klein *et al.*, 2022). Gellan gum or Phytagel^TM^, an alternative gelling agent, has been shown to enhance the growth of some slow-growing microorganisms (Takahashi *et al.*, 1992; Tamaki *et al.*, 2009; Nakamura *et al.*, 2011; Klein *et al.*, 2022). It has also been successfully used in the cultivation of hyperthermophilic archaea from deep-sea vents (Nakagawa *et al.*, 2004). Additionally, the method for preparing solid media was found to affect the colony formation ratio. Previous studies demonstrated that autoclaving agar along with other medium components inhibited microbial growth, potentially due to hydrogen peroxide production (Tanaka *et al.*, 2014; Kato *et al.*, 2020).

Therefore, the objective of the present study was to establish a straightforward, effective solid medium cultivation technique for deep-sea vent chemolithoautotrophs without the need for specialized and expensive equipment. To achieve this, our approach involved two main experimental strategies: to validate the method, we cultivated laboratory strains of chemolithoautotrophs on solid media and quantified the rate of colony formation, and then incubated a deep-sea vent sample on solid media to corroborate the effectiveness of our proposed method.

## Materials and Methods

### Strains

In the present study, we selected three *Campylobacterota* as representative deep-sea hydrothermal vent chemolithoautotrophs: *Sulfurimonas autotrophica* OK10^T^ (Inagaki *et al.*, 2003), *Sulfurovum* sp. NBC37-1, *Nitratiruptor* sp. SB155-2 (Nakagawa *et al.*, 2005a, 2005b, 2007), and one *Aquificota*: *Persephonella hydrogeniphila* 29W^T^ (Nakagawa *et al.*, 2003). *S. autotrophica* OK10^T^ is a strictly aerobic sulfur-oxidizing mesophile. *Sulfurovum* sp. NBC37-1 and *Nitratiruptor* sp. SB155-2 are capable of utilizing both hydrogen and sulfur compounds as an energy source. *P. hydrogeniphila* 29W^T^ is a strictly hydrogen-oxidizing thermophile. These strains were routinely cultivated in liquid-modified MMJHS medium under H_2_/CO_2_ (80:20) at temperatures of 25°C (*S. autotrophica* OK10^T^), 33°C (*Sulfurovum* sp. NBC37-1), and 55°C (*Nitratiruptor* sp. SB155-2 and *P. hydrogeniphila* 29W^T^). The original MMJHS medium comprised the following (L^–1^ of MMJ synthetic seawater): 1‍ ‍g NaNO_3_, 1‍ ‍g NaHCO_3_, 1‍ ‍g Na_2_S_2_O_3_·5H_2_O, and 10‍ ‍g elemental sulfur (Nakagawa *et al.*, 2005a; Nakagawa and Takai, 2006). The medium was modified in the present study by removing elemental sulfur and increasing thiosulfate to 3‍ ‍g to enhance the visibility of colonies and promote the growth of isolates.

### Solid medium preparations

The modified MMJHS medium was solidified using 1.5% (w/v) agar (Fujifilm Wako Pure Chemical) or 0.5% (w/v) gellan gum (Nacalai Tesque). Four different solutions (A to D) were prepared as follows: solution A contained double-concentrated MMJ synthetic seawater; solution B, 3% (w/v) agar; solution C, 1% (w/v) gellan gum; solution D, 5% (w/v) NaHCO_3_ and NaNO_3_ and 15% (w/v) Na_2_S_2_O_3_·5H_2_O. Using these solutions, we prepared four different types of solid media for evaluation: (1) “agar-autoclaved” medium prepared by aseptically mixing the same volume of autoclaved solutions A and B, (2) “agar-filtered” medium prepared by mixing the same volume of filter-sterilized solution A and autoclaved solution B, (3) “gellan gum-autoclaved” medium prepared by mixing the same volume of autoclaved solutions A and C, and (4) “gellan gum-filtered” medium prepared by mixing the same volume of filter-sterilized solution A and autoclaved solution C. Before solidification in a Split Petri Dish (4 sections) (VMR International), a 1/100 volume of filter-sterilized solution D was added to all media. The production of hydrogen peroxide in solid media was measured as previously described (Tanaka *et al.*, 2014). Additionally, *Nitratiruptor* sp. SB155-2 was inoculated on the modified MMJHS medium solidified with 1.5% (w/v) washed-agar. In this “agar-washed” medium, agar was washed in milli-Q water by sonicating for 60‍ ‍min and recovered by filtration (filter paper with a pore size of 10‍ ‍μm).

### Evaluation of solid media

Bacterial inocula were prepared from cultures grown in liquid-modified MMJHS medium for 24 h. Cells were then suspended in‍ ‍filter-sterilized MMJ synthetic seawater at concentrations varying between 10^4^–10^8^‍ ‍cells‍ ‍mL^–1^. Each dilution series was spot inoculated on solid media using 1‍ ‍μL of the suspension (10–10^5^ cells per spot) in four or five replicates across all sections of the Petri dish. After the inoculation, dishes were placed in a pouch bag‍ ‍(Mitsubishi Gas Chemical) along with one packet of AnaeroPouch^TM^-MicroAero (Mitsubishi Gas Chemical). The pouch bag was then flushed and filled with a mixture of filter-sterilized H_2_/CO_2_ at different ratios (80:20 or 50:50‍ ‍[v/v]). A gas mixture of H_2_/CO_2_ (25:75) was also tested for mesophilic strains. To prevent the desiccation of media, a piece of autoclaved KimWipe moistened with milli-Q water was included in the pouch. Additionally, in the case of incubations at 55°C, pouch bags were further enclosed with a Ziploc plastic bag. The pH of solid media under each gas phase was measured using a compact pH meter (AS-212; Horiba). Briefly, each solid medium was homogenized by pushing it through a needle hole, then pouring it into a Petri dish, and placing it in a pouch bag. Media were incubated under the respective H_2_/CO_2_ gas conditions for 24 h. After the incubation, homogenized media were removed from the pouch bag and promptly placed on the pH meter for measurements. Cultures were incubated for a minimum of 12 days at the temperatures described above. During this period, images of media were captured every 12 or 24‍ ‍h. *Sulfurovum* sp. NBC37-1 and *Nitratiruptor* sp. SB155-2 were inoculated on the “agar-autoclaved” medium and incubated in GasPak^TM^ jars (BD BBL) under an N_2_ atmosphere for comparisons.

### Environmental sample cultivation on solid media

A slurry sample of an active chimney structure was used as an inoculum, which was collected from the Noho site of the Sakai field (27°31.386'N, 126°59.209'E) in the Mid-Okinawa Trough, Japan, at a depth of 1,550‍ ‍m by means of the ROV Hyper-Dolphin (Dive#1860) during the JAMSTEC cruise NT15-13 in 2015 (Muto *et al.*, 2017). The sample was inoculated in duplicate onto both “agar-filtered” and “gellan gum-filtered” media. The gas phases tested were H_2_/CO_2_ (80:20) and H_2_/CO_2_ (50:50). Dishes were incubated at 55°C for 2–3‍ ‍weeks. Colony formation was monitored daily, and colonies were selected for direct PCR of 16S rRNA genes using the primers 27F and 1492R (Lane, 1991), as previously described (Takai *et al.*, 2001). Sanger sequencing was performed by Macrogen. The resulting sequences were assembled using GeneStudio ver 2.2.0.0 (GeneStudio) and compared to existing databases via the BLAST search algorithm (Altschul *et al.*, 1990). The 16S rRNA sequences obtained were deposited in the GenBank/EMBL/DDBJ databases under accession numbers LC775602–LC775696.

## Results and Discussion

### Cultivation in anaerobic jars

Neither *Sulfurovum* sp. NBC37-1 nor *Nitratiruptor* sp. SB155-2 were able to form colonies on the “agar-autoclaved” medium under the N_2_ atmosphere in GasPak^TM^ jars (data not shown), a typical cultivation strategy for anaerobes. Although deep-sea vent-dominating chemolithoautotrophs generally utilize both H_2_ and sulfur compounds as electron donors, their growth is often limited when relying solely on sulfur compounds (Nakagawa *et al.*, 2005b). Therefore, in the present study, we used pouch bags filled with H_2_-containing gas to cultivate these microorganisms. These bags are not only cost-efficient and space-saving, their physical flexibility also facilitates the easy replacement/control of the gas phase.

### Growth on solid media

**Fig. 1.**
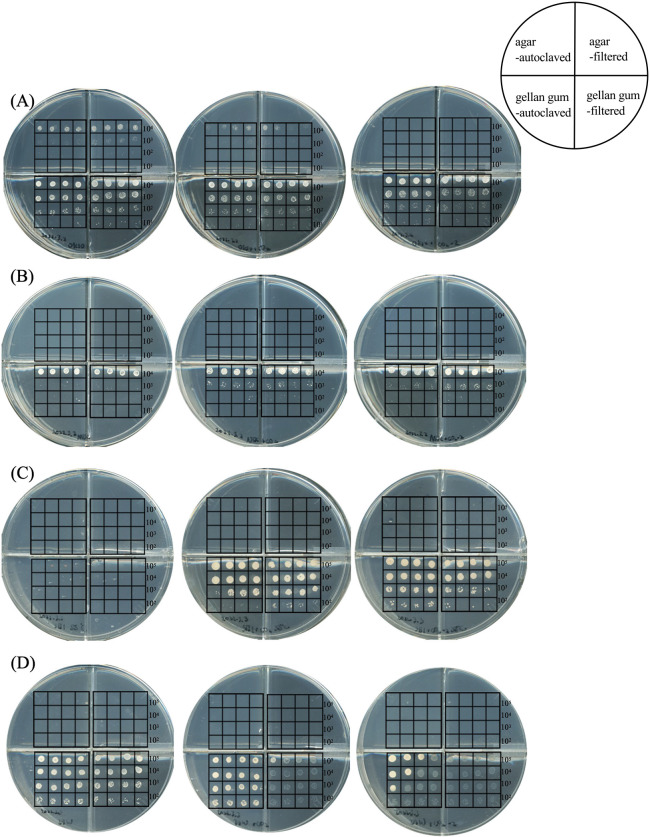
Growth of laboratory strains on solid media. (A) *Sulfurimonas autotrophica* OK10^T^ incubated at 25°C for 84 h; (B) *Sulfurovum* sp. NBC37-1 incubated at 33°C for 180 h; (C) *Nitratiruptor* sp. SB155-2 incubated at 55°C for 132 h; (D) *Persephonella hydrogeniphila* 29W^T^ incubated at 55°C for 60 h. In each strain, there were three dishes organized by CO_2_ concentrations from left to right (20%, 50%, and 75% [v/v]). In each dish, the upper left and right sections contained agar-autoclaved and agar-filtered media, respectively, while the lower sections included gellan gum-autoclaved (left) and gellan gum-filtered (right) media. The number of inoculated cells per spot, ranging from 10 to 10^4^, is indicated on the right side of the dish, with each being replicated four times. Refer to Movie S1 for corresponding time-lapse visuals.

An overview of the growth of each strain on solid media is shown in Fig. 1 and Movie S1. All strains exhibited faster growth, even at more diluted inoculation spots, on gellan gum-solidified media (shown in the lower sections) than on agar-solidified media (shown in the upper sections). Only *S. autotrophica* OK10^T^ grew on agar-solidified media, and growth was faster on the agar-filtered medium than on the agar-autoclaved medium (Fig. 1A and Movie S1). It is important to note that only this strain was originally isolated using an agar-solidified medium (Inagaki *et al.*, 2003), whereas the others were obtained through the dilution-to-extinction technique with liquid media. Although characterized as strictly aerobic, *S. autotrophica* OK10^T^ exhibited growth under these conditions. The medium contained no reductant, which implied that the strain utilized dissolved oxygen in the medium for respiration. Previous studies suggested that microbial growth on agar-solidified media was inhibited by the production of hydrogen peroxide (Tanaka *et al.*, 2014). However, in the present study, hydrogen peroxide was below the detectable limit in all the media tested (data not shown). This may have been due to thiosulfate in media serving as an energy source.

Since the H_2_/CO_2_ gas mixture was suggested to boost microbial growth on solid media, we further examined the effects of the gas phase composition on the growth of the strains by testing H_2_/CO_2_ (80:20), H_2_/CO_2_ (50:50), and H_2_/CO_2_ (25:75) (v/v). With the exception of *S. autotrophica* OK10^T^, all strains exhibited faster growth and notable progression at diluted inoculation spots under higher CO_2_ concentrations (Movie S1). Conversely, higher concentrations of CO_2_ inhibited the growth of *S. autotrophica* OK10^T^ (Fig. 1A and Movie S1). Although the underlying mechanisms have yet to be elucidated, some capnophilic members of *Campylobacterota* favor high CO_2_ concentrations (Bolton and Coates, 1983; Bury-Mone *et al.*, 2006). Given the CO_2_-rich nature of deep-sea vents coupled with deep-sea vent chemolithoautotrophs utilizing CO_2_ as their sole carbon source, it is conceivable that CO_2_ gas enhanced their growth. Another possible explanation for the effects of CO_2_ concentrations may be the pH of media and/or the CO_2_-HCO_3_^–^ equilibrium. The pH of media under H_2_/CO_2_ (80:20), H_2_/CO_2_ (50:50), and H_2_/CO_2_ (25:75) were 6.5, 6.0, and 5.7, respectively. For comparison, pH ranges for the growth of the tested strains were as follows: 4.5–9.0 (optimum pH 6.5) for *S. autotrophica* OK10^T^ (Inagaki *et al.*, 2003), 5.3–7.5 (optimum pH 6.0) for *Sulfurovum* sp. NBC37-1 (unpublished data), 5.2–7.5 (optimum pH 6.0) for *Nitratiruptor* sp. SB155-2 (unpublished data), and 5.5–7.6 (optimum pH 7.2) for *P. hydrogeniphila* 29W^T^ (Nakagawa *et al.*, 2003). Since the pH of media fell within the growth pH ranges of all these strains, pH as a single parameter cannot solely account for the variations observed in colony formation properties. Within these pH ranges, as the pH of media decreased, dissolved HCO_3_^–^ and CO_2_ concentrations were expected to decrease and increase, respectively. Consequently, the availability of dissolved CO_2_ at the surface of solid media may increase at elevated CO_2_ gas concentrations (Berg, 2011). It‍ ‍currently remains unclear whether *Aquificota* and *Campylobacterota* use CO_2_ or HCO_3_^–^ as a carbon source for the reductive TCA cycle; however, the CO_2_/HCO_3_^–^ ratio, depending on the pH of media and CO_2_ concentrations in the gas phase, may have affected their carbon fixation rate and growth.

### Effects of the gelling agent, sterilization method, and gas phase composition on the colony formation rate

**Fig. 2.**
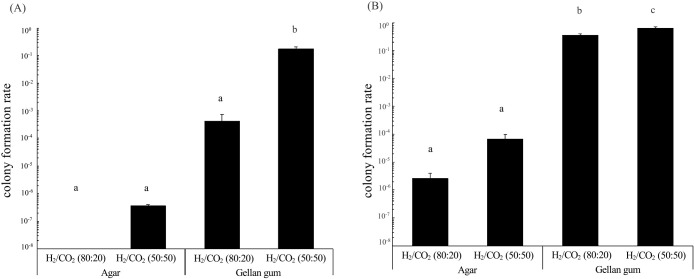
Colony formation rates of *Nitratiruptor* sp. SB155-2 (A) and *Persephonella hydrogeniphila* 29W^T^ (B) on agar- or gellan gum-filtered media. Rates were calculated by dividing the colony count by the inoculated cell count. Each bar represents the average colony formation rate, with standard errors (*n*=5). Distinct letters indicate significant differences (Tukey’s HSD, *P*<0.05). Data on colony formation rates on gellan gum-autoclaved media are available in supplementary Fig. S1.

To quantitatively evaluate the effects of gelling agents, sterilization methods, and CO_2_ concentrations, we measured the colony formation rates (the ratio of the colony count to the number of inoculated cells) of *Nitratiruptor* sp. SB155-2 and *P. hydrogeniphila* 29W^T^. We selected these strains because of their rapid and consistent colony formation. No significant differences were observed in colony formation rates between autoclaved (Fig. S1) and filtered (Fig. 2) media (*P*>0.1 by the Student’s *t*-test). The colony formation rate slightly decreased in the order of gellan gum under H_2_/CO_2_ (50:50), gellan gum under H_2_/CO_2_ (80:20), agar under H_2_/CO_2_ (50:50), and agar under H_2_/CO_2_ (80:20) (Fig. 2 and S1).

Under identical gas phases, the colony formation rates of *Nitratiruptor* sp. SB155-2 and *P. hydrogeniphila* 29W^T^ were higher on gellan gum-solidified media than on agar-solidified media. Gellan gum-solidified media under H_2_/CO_2_ (50:50) gas resulted in a significantly higher colony formation rate for *Nitratiruptor* sp. SB155-2 than the other conditions tested, achieving the highest colony formation rate of 1.7×10^–1^ (Fig. 2A). Similarly, *P. hydrogeniphila* 29W^T^ showed a higher colony formation rate on gellan gum-autoclaved medium under H_2_/CO_2_ (50:50) (Fig. S1B). Gellan gum-filtered medium under H_2_/CO_2_ (50:50) gas showed the highest colony formation rate for *P. hydrogeniphila* 29W^T^, reaching up to 6.3×10^–1^ (Fig. 2B). When combined with gellan gum and a gas phase of H_2_/CO_2_ (50:50), the colony formation rate of these strains was at least 10^5^-fold higher than the lowest colony formation rate. These results suggest that gelling agents and CO_2_ concentrations exerted stronger effects on the colony formation rate than sterilization methods, and also that gellan gum was advantageous for the cultivation of both *Nitratiruptor* sp. SB155-2 and *P. hydrogeniphila* 29W^T^. Furthermore, CO_2_ gas concentrations affected the colony formation rates of both *Nitratiruptor* sp.‍ ‍SB155-2 and *P. hydrogeniphila* 29W^T^, particularly *Nitratiruptor* sp. SB155-2, the colony formation rate of which markedly increased. As shown in the time-lapse movie (Movie S1) and colony formation rates (Fig. 2 and S1), the growth of *Nitratiruptor* sp. SB155-2 and *P. hydrogeniphila* 29W^T^ was enhanced by higher CO_2_ concentrations. A detailed ana­lysis, similar to transcriptome studies, is required to obtain a more detailed understanding of growth patterns on solid media and the effects of CO_2_ concentrations on colony formation.

### Effects of agar washing on the colony formation rate of *Nitratiruptor* sp. SB155-2

*Nitratiruptor* sp. SB155-2 was cultivated on both unwashed- and washed-agar media. The colony formation rate was higher on washed-agar media than on unwashed-agar media, at 5.4×10^–5^ and 3.6×10^–7^, respectively. Despite the higher colony formation rate facilitated by washing the agar, the rate on gellan gum media, which was 1.7×10^–1^ (Fig. 2), still surpassed that on washed-agar medium. Previous studies indicated that the suppression of colony formation on agar-solidified media was attributed to the presence of swarming inhibitors in commercial agar powders (Hara *et al.*, 2012) or the production of hydrogen peroxide when autoclaving agar with phosphate (Tanaka *et al.*, 2014). In the present study, we demonstrated that delayed growth (Fig. 1 and Movie S1) and low colony formation rates on agar-solidified media (Fig. 2) were at least partly due to water-soluble inhibitors in commercial agar powder. These inhibitors may be easily removed through simple water washing. Further research is needed to identify these specific inhibitors and clarify the mechanisms impeding the growth of chemolithoautotrophic bacteria on agar media.

### Cultivation of a deep-sea sample

**Fig. 3.**
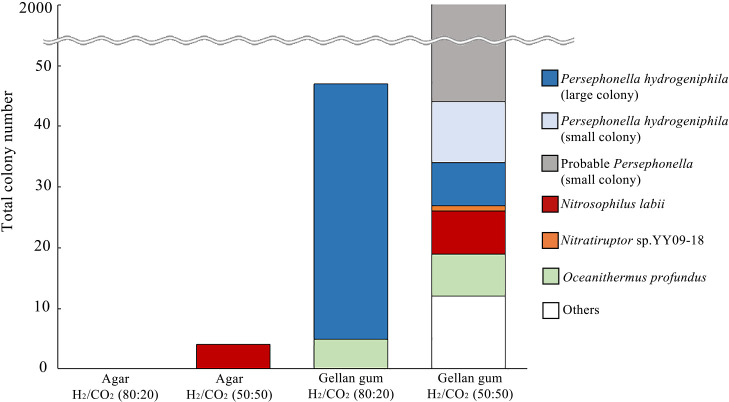
Phylogenetic property of colonies formed from a deep-sea hydrothermal chimney structure on agar- and gellan gum-solidified media. The gas phase was H_2_/CO_2_ (80:20) or H_2_/CO_2_ (50:50). Additional details on isolates are provided in supplementary Table S1.

A deep-sea hydrothermal vent sample was inoculated on solid media to assess the effects of gelling agents and the gas phase composition on environmental microbial communities. We inoculated 100‍ ‍μL of chimney slurry, equivalent to approximately 20‍ ‍mg (wet weight) of the chimney structure. Previous RNA-based ana­lyses revealed the dominance of diverse *Campylobacterota* in this sample (Muto *et al.*, 2017). On the agar-solidified medium, no colonies were observed under H_2_/CO_2_ (80:20), whereas four colonies developed under the H_2_/CO_2_ (50:50) atmosphere after a 14-days incubation at 55°C (Fig. 3). These colonies showed at least 99% similarity in their 16S rRNA gene sequence to *Nitrosophilus labii*, a moderately thermophilic chemolithoautotroph of the phylum *Campylobacterota*, originally isolated from the East Pacific Rise (Shiotani *et al.*, 2020).

Numerous colonies formed on gellan gum-solidified media, as previously reported for heterotrophic bacteria from soil and freshwater habitats (Janssen *et al.*, 2002; Sait *et al.*, 2002; Davis *et al.*, 2005; Tamaki *et al.*, 2005, 2009; Tanaka *et al.*, 2014; Kato *et al.*, 2018). Forty-seven colonies were grown on gellan gum media under H_2_/CO_2_ (80:20), displaying at least 98% similarity in their 16S rRNA gene sequences to *P. hydrogeniphila* (42 isolates) (Nakagawa *et al.*, 2003; Mino *et al.*, 2013) and *Oceanithermus* (5 isolates) (Miroshnichenko *et al.*, 2003). Notably, no *Campylobacterota* colonies were found under these cultivation conditions (Fig. 3 and Table S1). The gellan gum-solidified medium under a H_2_/CO_2_ (50:50) gas phase showed the highest colony-forming capacity, yielding more than 2,000 colonies of various sizes (Fig. 3). Large colonies (>1‍ ‍mm in diameter) were identified as *Nitrosophilus* (7 colonies), *Nitratiruptor* (1 colony) (Nakagawa *et al.*, 2005c), *Persephonella* (7 colonies) (Mino *et al.*, 2013), *Oceanithermus* (7 colonies), *Kyrpidia* (8 colonies) (Reiner *et al.*, 2018), *Bacillus* (3 colonies), and *Aeribacillus* (1 colony) (Table S1). The growth of strictly heterotrophic bacteria, *i.e.*, *Bacillus*, *Aeribacillus*, and *Oceanithermus* (Nakamura *et al.*, 1988; Miroshnichenko *et al.*, 2003; Minana-Galbis *et al.*, 2010), on gellan gum media may be attributed to the use of gellan gum as an energy and/or carbon source. It is important to note that the development of these heterotroph colonies was slower than that of autotroph colonies.

Given their morphological similarity, ten of the more than 2,000 small colonies were examined. Each colony was highly similar to *P. hydrogeniphila* (Table S1), suggesting that the remaining colonies also belonged to the genus *Persephonella*. This is consistent with enhanced colony formation by a *Persephonella* laboratory isolate on gellan gum media under higher CO_2_ concentrations. However, we observed an inconsistency where although *P. hydrogeniphila* 29W^T^ showed a higher colony formation rate on gellan gum under H_2_/CO_2_ (80:20), small *Persephonella* colonies did not emerge from the chimney sample. This discrepancy may be attributed to the dormancy of *Persephonella* cells in the long-stored environmental sample, which may require higher CO_2_ concentrations for their revival and growth.

Although the taxonomic novelty of the isolates was not apparent, a large number of chemolithoautotrophs from the genera *Nitrosophilus*, *Nitratiruptor*, and *Persephonella* were obtained. These bacteria had only previously been cultivated in liquid media (Nakagawa *et al.*, 2005c; Mino *et al.*, 2013). Our simplified cultivation approach has facilitated their successful colony formation on solid media for the first time.

## Conclusion

The present study demonstrated that the cultivation of deep-sea vent chemolithoautotrophs on solid media may be optimized by using gellan gum and pouch bags filled with an H_2_/CO_2_ gas mixture, thereby eliminating the need for specialized equipment, such as anaerobic glove boxes. Although the detection of some heterotrophs warrants caution, this new straightforward approach effectively isolated chemolithoautotrophic microorganisms from deep-sea hydrothermal fields. While the present study did not yield phylogenetically novel chemolithoautotrophs, the increased efficiency in colony formation is significant for reasons beyond taxonomy. The ability of our method to yield a large number of isolates of the same species from a single sample enhances its utility in conducting biogeographic and ecological ana­lyses, marking it as a valuable tool. Furthermore, our method offers a practical technique for future research on deep-sea vent chemolithoautotrophs. Its efficiency in colony formation may accelerate the development of genetic modification procedures for these microorganisms.

## Citation

Muto, H., Miyazaki, J., Sawayama, S., Takai, K., and Nakagawa, S. (2023) A Simple and Effective Method for Solid Medium Cultivation of Strictly Hydrogen- and Sulfur-oxidizing Chemolithoautotrophs Predominant in Deep-‍sea Hydrothermal Fields. *Microbes Environ ***38**: ME23072.

https://doi.org/10.1264/jsme2.ME23072

## Supplementary Material

Supplementary Material 1

Supplementary Material 2
