# Supplementary material for: A Simple and Effective Method for Solid Medium Cultivation of Strictly Hydrogen- and Sulfur-oxidizing Chemolithoautotrophs Predominant in Deep-‍sea Hydrothermal Fields
Source: Microbes Environ. 2023 Dec 16;38(6):ME23072. doi: 10.1264/jsme2.ME23072 (PMC10728628; doi:10.1264/jsme2.ME23072)
Supplement: Supplementary file 1 — Supplementary Material 1 [file 38_23072_s1.pdf]

Fig. S1. Colony formation rates of *Nitratiruptor* sp. SB155-2 (A) and *P. hydrogeniphila* $29W^T$ (B) on agar- or gellan gum-autoclaved media. Each bar represents the average colony formation rate with standard errors. Different letters indicate statistically significant differences (Tukey's HSD, $p < 0.05$).

Movie S1. Time-lapse representation of microbial growth on solid-media. Showcased are: (A) *Sulfurimonas autotrophica* $OK10^T$ at 25 °C; (B) *Sulfurovum* sp. NBC37-1 at 33 °C; (C) *Nitratiruptor* sp. SB155-2 at 55 °C; (D) *P. hydrogeniphila* $29W^T$ at 55 °C.

Table S1. Summary of microorganisms grown on solid-media.

Representative strain	Other isolates	Closest match (Accession number)	Similarity (%)	Accession numbers
Nitrosophilus labii AC1	AC1-4, GC1-5, GC20-21	*Nitrosophilus labii* (AP022826.1)	99.7-100	LC775602-775605, LC775653-775657, LC775672-775673
Nitratiruptor sp. YY09-18 GC11	GC11	*Nitratiruptor* sp. YY09-18 (AP023065.1)	100	LC775663
Persephonella hydrogeniphila G1 (large colony)	G1-G13, G15-G17, G19-20, G23-46, GC10, GC13-14, GC28, GC35-36, GC44	*Persephonella hydrogeniphila* (NR_024797.1)	98.9-100	LC775606-775618, LC775620-775622, LC775624-775625, LC775628-775651, LC775662, LC775665-7766655, LC775680, LC775687-775688, LC77569696
Persephonella hydrogeniphila GC6 (small colony)	GC6-9, GC29-34	*Persephonella hydrogeniphila* (NR_024797.1)	98.6-99.2	LC775658-LC775661, LC775681-775686
Oceanithermus profundus G14	G14, G18, G21-22, G47, GC12, GC15-18, GC23, GC27	*Oceanithermus profundus* (NR_074604.1)	97.2-100	LC775619, LC775623, LC775626-775627, LC775652, LC775664, LC775667-775670, LC775675, LC775679
Kyrpidia spormannii GC22	GC22, GC37-43	*Kyrpidia spormannii* (LR792684.1)	98.2-99.5	LC775674, LC775689-775695
Bacillus smithii GC24	GC24-26	*Bacillus smithii* (CP012024.1)	99.9-100	LC775676-775678
Aeribacillus pallidus GC19	GC19	*Aeribacillus pallidus* (MG651190.1)	99.6	LC775671

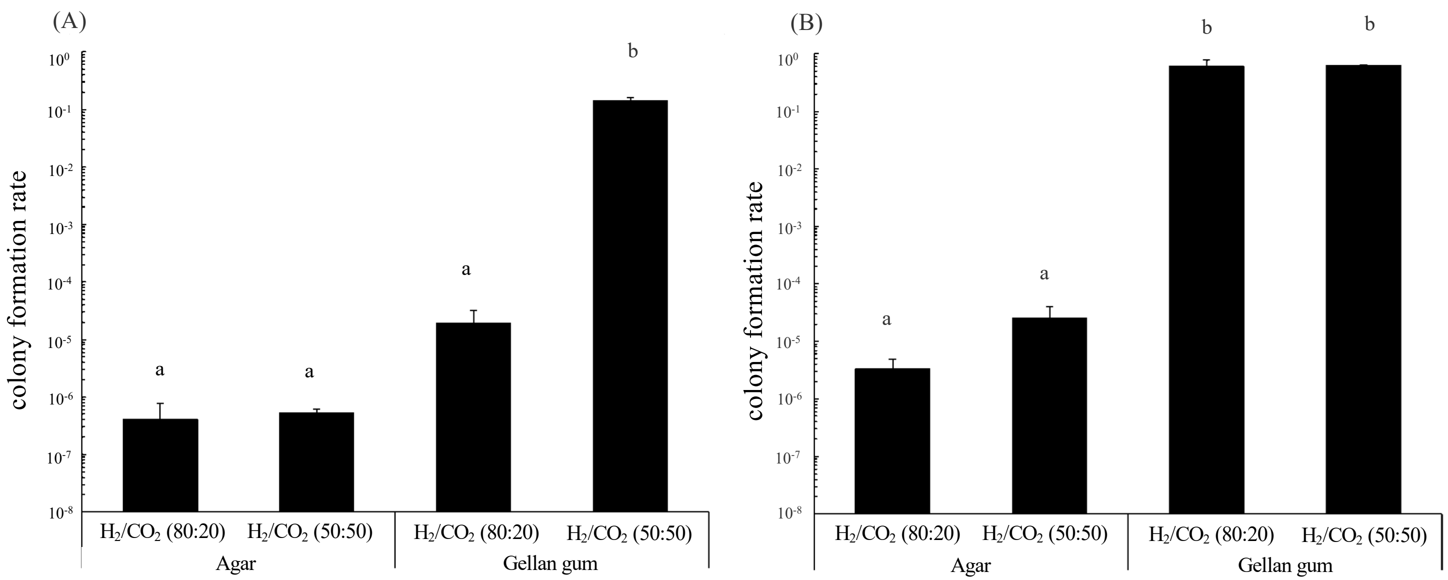
(A)
(B)
colony formation rate
10^{0}
10^{-1}
10^{-2}
10^{-3}
10^{-4}
10^{-5}
10^{-6}
10^{-7}
10^{-8}
a
b
H_2/CO_2 (80:20)
H_2/CO_2 (50:50)
Agar
Gellan gum

Fig. S1
